# The Rapidly Evolving Concept of Whole Heart Engineering

**DOI:** 10.1155/2017/8920940

**Published:** 2017-11-09

**Authors:** Laura Iop, Eleonora Dal Sasso, Roberta Menabò, Fabio Di Lisa, Gino Gerosa

**Affiliations:** ^1^Cardiovascular Regenerative Medicine Group, Department of Cardiac, Thoracic and Vascular Surgery, University of Padua, Padua, Italy; ^2^Venetian Institute of Molecular Medicine, Padua, Italy; ^3^Institute of Neuroscience, National Research Council (CNR), Padua, Italy; ^4^Department of Biomedical Sciences, University of Padua and Venetian Institute of Molecular Medicine, Padua, Italy

## Abstract

Whole heart engineering represents an incredible journey with as final destination the challenging aim to solve end-stage cardiac failure with a biocompatible and living organ equivalent. Its evolution started in 2008 with rodent organs and is nowadays moving closer to clinical application thanks to scaling-up strategies to human hearts. This review will offer a comprehensive examination on the important stages to be reached for the bioengineering of the whole heart, by describing the approaches of organ decellularization, repopulation, and maturation so far applied and the novel technologies of potential interest. In addition, it will carefully address important demands that still need to be satisfied in order to move to a real clinical translation of the whole bioengineering heart concept.

## 1. Historical Excursus

“In attempting to discover how much blood passes from the veins into the arteries I made dissections of living animals, opened up arteries in them, and carried out various other investigations. I also considered the symmetry and size of the ventricles of the heart and of the vessels which enter and leave them (since Nature, who does nothing purposelessly, would not purposelessly have given these vessels such relatively large size). I also recalled the elegant and carefully contrived valves and fibres and other structural artistry of the heart; and many other points. I considered rather often and with care all this evidence, and took correspondingly long trying to assess how much blood was transmitted and in how short a time. I also noted that the juice of the ingested food could not supply this amount without our having the veins, on the one hand, completely emptied and the arteries, on the other hand, brought to bursting through excessive inthrust of blood, unless the blood somehow flowed back again from the arteries into the veins and returned to the right ventricle of the heart. In consequence, I began privately to consider that it had a movement, as it were, in a circle” [1].

On 3 December 2017, an important finish line will be reached, that is, 50 years from the world's first human-to-human heart transplantation. This intervention was successfully realized by the pioneering cardiac surgeon Christiaan N. Barnard and now is a life-saving therapy for many patients with end-stage heart failure.

**Figure 1:**
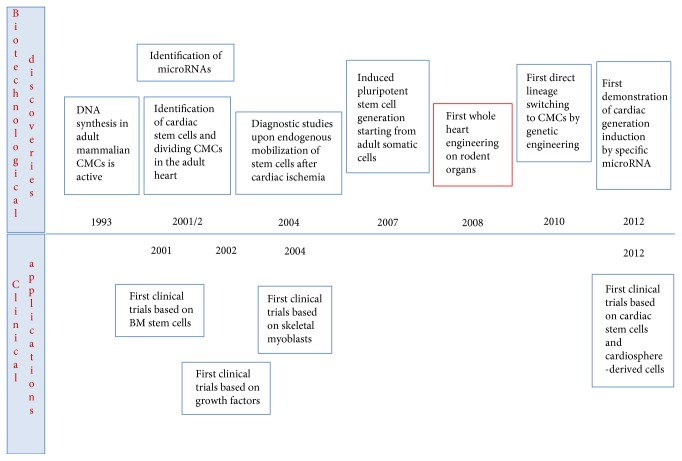
The most striking technological advancements and clinical applications anticipating the birth and evolution of the whole bioengineering heart concept (CMCs: cardiomyocytes; BM: bone marrow).

Among the causes leading to the failure of the cardiac organ, myocardial infarction appears as the most responsible. In order to block the progression and induce repair of the myocardial scar, several therapeutic approaches have been investigated thanks also to the technological advancements offered by cardiovascular regenerative medicine (Figure 1). This innovative biomedical branch is aiming since the 90s to propose biotechnological alternatives or adjuvant solutions to conventional pharmacological or surgical treatments, possibly stimulating the heart's endogenous regenerative properties. Mobilization of resident stem cell populations or administrations of exogenous progenitors have been pursued to antagonize the remodeling process leading to irreversible loss of cardiac tissue and hence to cardiac failure. Several growth factors and stem cells have been considered in clinical trials as potential therapies for acute or chronic cardiac ischemia; however, reported effects are still controversial and only partially beneficial for the global heart function.

Heart failure is a worldwide burden affecting approximately 26 million of patients [2, 3], with an incidence of 50,000–100,000 new severe cases diagnosed on annual basis.

For these patients, the uniquely biological therapy is represented by heart transplantation. Each year, 4000 interventions of cardiac transplant are performed [4, 5]; however, a dramatic mismatch exists between the numbers of treated patients and subjects remaining on the waiting list. Moreover, in half a century of this clinical practice, several drawbacks emerged as, in particular, the complications of immune response and its suppressive therapies [6].

In 2008, about 40 years after the successful heart transplant, another pioneering work was realized, that is, the decellularization of the whole heart, as initial mile stone for the development of a fully bioengineered substitute. In Nature Medicine, Ott et al. described for the first time the obtainment of a decellularized organ extracellular matrix (ECM) from the rat heart, also defined as the “ghost heart” [7]. The coronary arteries of a native rodent heart were perfused in antegrade direction with solutions based on detergents, until the resident cells were washed out leaving only the extracellular matrix of the organ.

This outcome appeared immediately promising because it offered potentialities to solve a recurrent controversial issue for tissue engineering approaches aiming at repairing heart damages through *in vitro* tissue equivalents, that is, the vascularization hurdle [8]. The possibility to rely on already existing vasculature network scaffolding might overcome the problem of core necrosis, generated when the tissue exceeds a thickness of 100 *μ*m and is not promptly vascularized.

Apart from this technological improvement, this work represents definitely the breakthrough to advance a more biocompatible and self-like solution for cardiac failure, using the obtained natural scaffold as starting matrix to engineer a heart with patient's cells.

In this review article, we will explore the evolution of this first attempt in terms of methodologies so far applied to generate acellular cardiac matrices and repopulate them, innovative ancillary techniques and questions that remain still to be considered for a clinical application of the whole heart engineering concept.

## 2. Methodologies for the Decellularization of the Heart

So far, the artificial reproduction of the complete spatial geometry, structural organization, and biological functionality of solid organ ECM is a challenging mission, even though in the current bioprinting technology era [9]. The solution to this demanding question is represented by decellularization procedures. These methods have to deal with two antithetic tasks: the achievement of the ideal natural ECM, endowed with biological activity and biomechanical competence, and the need for a complete removal of endogenous cellular components to avoid inflammatory events, immune rejection [10], and calcification of the scaffolds. Therefore, the optimization of cell disassembly and extraction has to meet necessarily the minimization of structural and functional impairment. This compromise might be achieved by a wise balancing of the critical issues in decellularization approaches: chemical selection, concentration, and exposure.

Whole organ decellularization procedures are commonly based on optimized combinations of physical, chemical, and enzymatic methodologies [11, 12]. In the case of the heart, the coronary system is directly used to convey the decellularization solutions, maximizing their penetration and diffusion through the full thickness of the cardiac wall, in a process called organ perfusion.

### 2.1. Heart Perfusion

The first perfused mammalian heart was achieved by Langendorff [13], according to a methodology still applied nowadays without substantial modifications. In this setup, blood or perfusion solution is delivered into the heart in a retrograde manner by inserting a cannula in the ascending aorta. Thus, the aortic valve is closed, and the perfusion buffer bypasses the left ventricle and enters in the coronary arteries through the ostia. The perfusion solution flows through the coronary circulation and reaches the right atrium via the coronary sinus. As perfusion starts, the heart recovers its own automaticity and beats spontaneously for many hours (as reviewed in [14]).

**Figure 2:**
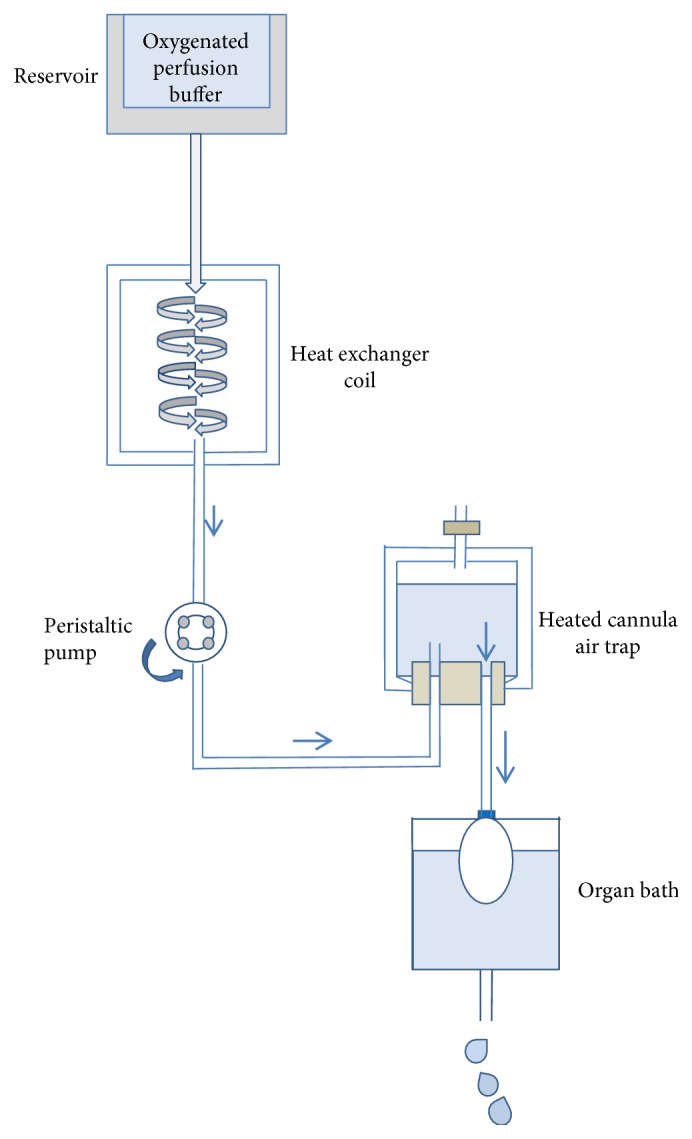
Schematic configuration of the Langendorff apparatus system as applied nowadays.

At present (Figure 2), isolated perfused heart preparations are largely based on adaptations of this first method [13]. Currently, the preparation involves the cannulation of the aorta of a heart harvested from an anesthetized animal. The organ is immediately immersed in a cold solution at 4°C and mounted on the perfusion apparatus. The latter is covered by a water jacket, maintained at 37°C by warm circulating water. The cannula is attached to the outflow of a reservoir containing the perfusion solution. This oxygenated solution is maintained at 37°C and continuously gassed with a mixture containing 5% CO_2_ and 95% O_2_, opportunely balanced to guarantee normal aerobic perfusion. The perfusion buffer is a physiological salt solution containing bicarbonate and mimics the ionic content of plasma, as defined by Krebs and Henseleit. The perfusion solution contains in mmol/l NaCl 118.5, NaHCO_3_ 25.0, KCl 4.7, MgSO_4_ 1.2, KH_2_PO_4_ 1.2, glucose 11.0, and CaCl_2_ 1.4 [15]. It is delivered into the aorta through the coronary arteries at 37°C in the presence of continuous gassing with 5% CO_2_ to yield a physiological pH of 7.4 [16–18].

As in the first experiments by Langendorff, the retrograde perfusion induces the closure of the aortic valve and no fluid can perfuse the left ventricular chamber. Finally, the solution is released through the coronary veins and right atrium, once all the cardiac tissues have been perfused [19].

Particular attention is addressed by the expert operator during the gentle cannulation of the aorta and the application of perfusion settings in order to prevent the induction of aortic valve incompetence.

Based on the experiment to be performed, the perfusion modality can be either set at constant hydrostatic pressure or at constant flow rate, thanks to the use of a calibrated roller pump. By maintaining the pressure unchanged during all the perfusion, it is possible to preserve the ability of the cardiac organ to regulate autonomously the vascular tone of the coronary arteries. Indeed, it is important to consider that the diameter of the vessel changes, especially in pathophysiological conditions. Conversely, a constant flow perfusion is more adapt to simulate ischemic conditions, also characterized by low flow, as well as to investigate modifications in the coronary vasomotor tone induced by vasoactive molecules [20, 21]. Therefore, it appears essential to rely on a Langendorff system integrating both perfusion modalities, especially in the case of protocols applied to obtain primary cultures of cardiac cells.

Independently from the perfusion modality, the use of the Langendorff model offers several advantages in terms of flexibility (size and species of the organ), reproducibility, and cost-effectiveness, so that its application can foster translational outcomes without the bias introduced by the presence of other organs or core necrosis [22, 23].

In addition, its configuration might be highly versatile with benefits for several applications, as biochemical studies, electrophysiological characterization, metabolic tests, and pharmacological assays. Besides, it can be combined with other analyses, as microscopic evaluations on biopsies.

Langendorff apparatus appears very attractive also for the whole decellularization of mouse, rat, porcine, and human hearts. To this end, adapted systems are prevalently used at constant hydrostatic pressure, even if combined modalities are also applied [7, 24, 25].

### 2.2. Decellularization Agents

Decellularization solutions are prevalently based on different concentrations of ionic, such as sodium dodecyl sulfate (SDS) and deoxycholic acid (DCA), and nonionic detergents, such as Triton X-100. Trypsin is often used as enzymatic component combined with ethylenediaminetetraacetic acid (EDTA), as chelating agent. Osmotic shocks, as well as freezing steps and agitation, are introduced to facilitate cellular membrane disruption. The use of nucleases to remove residues of nucleic acids is adopted, but sporadically.

A schematic description of the different methodologies applied so far for heart decellularization can be found in Table 1.

As mentioned before, the first protocol for whole heart decellularization has been published by Ott et al. in 2008 [7]. In this study, four different decellularization procedures were compared. Two of these methods were based on ionic and/or nonionic detergents (SDS and Triton X-100, resp.), one on enzymes and the last one on polyethylene glycol (PEG). The combination of 1% SDS and 1% Triton X-100 demonstrated to be the most effective one. A full decellularization was achieved, allowing the removal of 96.7% of native DNA. No contractile elements or other cellular debris could be identified by histological and scanning electron microscopy analyses. Furthermore, the cardiac ECM retained its structural organization with preserved distribution of collagen type I, collagen type III, and fibronectin. The integrity of vascular and endocardial basement membranes, as well as cardiomyocyte basal lamina, was demonstrated.

Two years later, in 2010, the decellularization of whole heart was scaled up to porcine organs by Wainwright et al. [26]. The protocol introduced the use of freezing/thawing to facilitate cellular lysis prior to detergents steps. Then, the decellularization was carried on by means of osmotic shocks, enzymes, acids, and surfactants. Chelating agents (0.05% EDTA), 0.02% trypsin, 3% Triton X-100, and 4% DCA were coupled with low concentrations of a biocide [12] (0.05% sodium azide). Despite only 10 hours were needed to accomplish the full protocol, a 92% reduction of DNA was estimated. Evaluations performed by histology and immunohistochemistry confirmed the complete cell removal and no damage of collagen types I, III, and IV, and elastin.

Unfortunately, the outcomes obtained by Ott (protocol I) and Wainwright (protocol II) were not confirmed by Akhyari et al. [24]. Both protocols resulted in incomplete decellularization of rat hearts with retention of cellular residues, as basophilic elements (DNA removal was about 43% and 80%, resp.). These results were compared with other two methodologies that were able to achieve a greater DNA reduction (more than 95%). The first one (protocol III) was transposed from peripheral nerve decellularization, and, besides sodium azide, EDTA, SDS, DCA, and Triton X-100 in concentration already reported, it added the use of 20% glycerol as dehydrating agent. The second one (protocol IV) was newly developed by the authors and introduced the use of saponin, combined with 1% SDS, 1% DCA, 0.05% sodium azide, 20% glycerol, and 25 mM EDTA.

Weymann et al. proposed a protocol for porcine heart decellularization, based only on 4% SDS administered for 12 h at 37°C to increase its extraction efficacy. By the increased concentration of this ionic detergent, the complete cell elimination was achieved with the preservation of ECM architecture and distribution. However, the decellularized heart maintained 82% of the native DNA [25, 27].

During the decellularization process, high stresses might be induced through the coronary wall. In order to prevent potential damages, the progressive increase of the flow-controlled perfusion was proposed by Remlinger et al. for porcine organs [28]. Basically, Wainwright's protocol was revisited increasing trypsin concentration (from 0.02% to 0.2%) and adding extensive washes. The results confirmed the complete decellularization and an improved DNA removal.

Other four protocols based on the use of Triton X-100, EDTA and trypsin were proposed by Merna et al. [29]. The comparison was completely based on optical imaging techniques (multiphoton microscopy and image correlation spectroscopy). The results demonstrated that, in respect to Triton X-100, the prolonged use of trypsin gradually induced the loss of collagen crimping. On the opposite, elastin distribution appeared to be preserved with the Triton X-100-based protocol. However, only the combination of the two decellularization reagents was able to assure the best DNA removal (almost 91%).

The decellularization attempt by Methe et al. was demonstrated to be ineffective. In fact, the decellularization was not achieved even after 8 cycles of 4% sodium deoxycholate (SDC) and 1% Triton X-100 solutions interposed by 6 hours of washes. Masson's trichrome evidenced the presence of almost intact myocardial bundles, while contractile units were visible with transmission electron microscopy evaluation [30].

Momtahan et al. adapted Ott's protocol to pig hearts using a customized decellularization system. Perfusion times of SDS and Triton X-100 were increased to 6 and 12 hours, respectively. The automation of the pressure control, already successful in other settings, allowed the shortening of SDS perfusion period, the elimination of almost 98% of native DNA, and of the other cellular components, while good preservation of the ECM was reached [31].

Kitahara et al. performed an additional reduction of SDS and Triton X-100 perfusion times. In this case, the process of cellular disassembly was probably facilitated by the initial freezing/thawing of the porcine heart [32].

Finally, the transition to human hearts took place in 2016 when Ott's group, in collaboration with the New England Tissue Bank, decellularized human organs not suitable for transplantation [33]. The used protocol was a further adaptation of the original one proposed by the same Ott et al., but in this experiment, 1% SDS and 1% Triton X-100 were perfused for 168 and 24 hours, respectively. For human heart decellularization, a shorter variation was eventually proposed, as based only on perfusing 60 liters of 1% SDS for 4 days [34].

Ultimately, Pati et al. introduced a further step in Ott's original protocol, that is, a treatment based on 0.1% peracetic acid and 4% ethanol, able to assure a decontamination of the decellularized porcine heart scaffold [35].

## 3. Biochemical, Biomechanical, and Bioinductive Properties of Decellularized Hearts

The peculiar architecture of biological matrices is constituted mostly by few molecules with both functional and structural properties: several types of collagens, fibronectin, laminin, elastin, GAGs, and growth factors [36]. Biochemical, biomechanical, and bioinductive properties of decellularized scaffolds have been purposely developed, during the evolution, to guarantee the inevitable “dynamic reciprocity” with resident cells [37]. Therefore, the ECM integrity is fundamental to regulate the constructive remodeling when used as scaffolds for the regeneration of tissues and organs [38].

### 3.1. Biochemical Properties

In most of the protocols for whole heart decellularization, the evaluation of the effective cell extraction has been followed by the biochemical quantification of predominant ECM components. Each method of decellularization is potentially associated with ECM disruption or integrity loss. SDS, Triton X-100, and a combination of trypsin/EDTA might cause reduction of GAG concentration. Ionic detergents are responsible for the breakdown of proteins, such as structural collagen and basement membrane components. Elastin can be damaged by the use of enzymatic methods, while laminin and fibronectin are more sensitive to the action of nonionic detergents [11].

Generally, insoluble collagen demonstrated to be highly preserved independently on the tested protocol, while the soluble form, more delicate and immature, decreased and appeared to be more preserved in the right side of the heart [30, 31, 33]. Only in case of partial decellularization, the amount of these two types of collagens was not statistically different from the one evaluated in native conditions [30].

Akhyari et al. specifically quantified the amount of collagen I by means of Western blot. The structural protein resulted increased in the decellularized tissue, with respect to the control quantity [24], an artifact that is probably depending on the normalization to corresponding varied dry weights.

Regarding the quantification of GAGs, the variations were not homogeneous among the protocols. In some cases, a slight increase was registered, while in others the opposite. Nevertheless, no significant differences were demonstrated with respect to the control groups in most of the procedures [7, 26, 27, 30]. Significant loss of GAGs was reported in porcine decellularized right ventricle, right atrium, and septum after the use of SDS and Triton X-100 [31] and in case of human donors passed away from noncardiac death [33]. A dramatically high reduction of GAGs (about 70%) was reported following the introduction of saponin as decellularization agent [24].

Analogous observations were disclosed regarding the elastin quantification. It resulted decreased, but not significantly, in respect to the cadaveric ECM, except in the case of human hearts decellularized using SDS [33]. Moreover, elastin amount, as well laminin quantity, appeared to be more preserved in Western blot analysis, if SDS-based decellularization was performed instead of combinations of DCA, glycerol, EDTA, trypsin, and saponin [24].

Proteomic analyses carried on decellularized human scaffolds demonstrated that the most preserved proteins during the process are the prevalent constituents of ECM (matrisome), that is, collagens, laminins, fibrillins, and proteoglycans. However, the entire proteome was reduced of 89% by the extensive SDS-based protocol [33].

### 3.2. Biomechanical Features

The parenchyma is particularly abundant in the heart and implicates the presence of a thin and limited stroma. However, the ECM does not play a marginal role in cardiac mechanics. De facto, the working myocardium cannot be considered as a continuum because the muscular fibres are organized in *laminae* [39]. Highly organized bundles of collagen connect muscular fibres, adjacent cardiomyocytes, and cardiomyocytes-capillaries. They are responsible for load bearing and prevent the tissue failure by limiting the relative fibre slipping [40]. Moreover, tissue biomechanics has a biological role as well, because the scaffold stiffness might affect the correct maturation and differentiation following cellular engraftment [41].

After the decellularization procedures, the loss of cardiomyocytes is directly translated in loss of volume. This effect induces the collapse of the cardiac wall and tissue compactness. Biomechanical tests performed on decellularized heart wall might be useful for a comparative evaluation of the biomaterial before and after decellularization.

Uniaxial and biaxial tensile tests are the easiest mechanical tests to perform. Moreover, they allow collecting several information about the elasticity and the failure properties of the tissue.

Ott et al. performed equibiaxial testing, by stretching the cross-shaped left ventricles at 40% of deformation. Fibrin gel was adopted as control. The circumferential direction of decellularized samples resulted stiffer than the longitudinal one, while elastic modulus of the control was significantly smaller compared to the other conditions [7].

A controlled biaxial load of 20 kPa was applied to square samples of native and decellularized left human ventricles. This test confirmed the maintenance of the anisotropy in treated samples, without any statistical difference to the control. The variability of the mechanical behavior in the two considered directions (longitudinal and circumferential) was not statistically significant for both conditions [33].

Inflation and compression tests are also applied to evaluate biomechanical features of decellularized heart. Weymann et al. used a liquid-filled latex balloon, connected to a manometer, to test the pressure response of the left ventricle in function of different ventricular volumes. The resulting curve presented strong similarity with the control one, differently from those generated by using the Wainwright's protocol [25–27]. Wainwright et al. performed ball burst biomechanical test following the ASTM D3787-07 International standards to compare right and left ventricles. No statistically significant differences were identified considering the extensibility of the tissue [26].

A progressive decrease of the compression modulus during the decellularization protocol was observed upon combination of EDTA, trypsin, and Triton X-100, while Triton X-100 alone determined its increase of 150%. This observation is confirmed by the structural reorganization of collagen bundles that appeared highly crimped and compact after cell removal [29].

After SDS and Triton X-100 decellularization by low pressure, Momtahan et al. confirmed the ventricle reduction of the compression modulus in respect to the native control [31].

### 3.3. Bioinductive Properties

During postinjury regeneration in nonamniotic vertebrate species, as salamanders, newts, axolotls, and zebrafish, a blastema tissue is formed in a dedifferentiation-induction process [42], also described as epimorphic regeneration. Interestingly, this process requires the activation of genes regulating cardiac cell proliferation and ECM degradation, but not its synthesis [43]. In addition, an incomplete adaptive immunity reduces the immunoinflammatory response in the regenerating myocardium. Macrophages are able to control fibroblast conversion to myofibroblasts, thus preventing scar formation [44].

Conversely, in mammals, repair is characterized by upregulation of genes correlated to ECM synthesis and immune response in a tissue particularly rich in fibroblasts [42].

In this perspective, the ECM might be perceived as a barrier to tissue regeneration, introduced during animal evolution. In other words, the fibres of the mammalian ECM behave as support and anchorage, likely regulating the maintenance of the differentiated cell phenotype. The different ratio of cells and ECM in mammalian tissues is therefore particularly essential to safeguard heart pump function, at the expense of the possibility of regeneration.

As previously described, the ECM of the mammalian heart is a very intricate network of prevalently collagens, elastin, glycosaminoglycans, and glycoproteins with a specific distribution and orientation. Cardiac ECM proteins are therefore organized in a complex hierarchy, difficult to be established by the artificial assembly of biomaterials. The use of a decellularized heart scaffold is conversely advantageous because recellularization will happen into a native and mature ECM. The ECM exercises its bioactivity on contacting cells through several signaling modalities, that is, matrikines, mechanotransduction, and binding to growth factors.

The cardiac matrisome strictly influences the phenotype of resident cells in native tissues.

Remarkably, specific domains are repeated in the protein sequence of ECM elements following a peculiar organization [45]. Matrikines derived from decellularized heart tissues or exogenously introduced have been proven to induce the differentiation of stem cells into mature, contractile cardiomyocytes, smooth muscle cells, and/or endothelial cells [46–50]. ECM proteins, as fibronectin or laminin, have been found in the cardiac niche for the maintenance of stemness and secreted after myocardial injury as a guide for tissue repair [51, 52].

Mechanosensing is also a potent driver of stem cell conversion. The interaction between integrins and cell actin cytoskeleton in cardiomyocytes is able to activate kinase molecular pathways inducing the formation of focal adhesions. These binding elements are at the base of the mechanoelectrical coupling, so important for the working myocardium (as reviewed in [53]). Scaffold stiffness and exposure to the changes of mechanical forces during the cardiac cycle play also a fundamental role in mechanosensing-related cell differentiation [54].

Matrikines and mechanotransduction proteins must therefore be well preserved in distribution and integrity in the hearts submitted to decellularization in order to stimulate effective differentiation of seeded cells.

In the native heart, growth factors are found associated with the ECM. In particular, heparan sulfate is a glycosaminoglycan, retrieved in the pericellular spaces and ECM, and is known to be involved in the regulation of heart development and angiogenesis, as well as in disease, thanks to its interaction with several growth factors, for example, bFGF, VEGF, and HB-EGF [55–57].

Methe and colleagues evaluated the content of angiogenic growth factors still withheld after heart decellularization. The application of Luminex technology to native and decellularized auricular and ventricular tissues evidenced that in acellular scaffolds, there was no significant change in the amount of VEGF-A and C, IL-8, leptin, and FGF-1, while a decrease was observed for other forms of VEGF, bFGF, angiopoietin-2, bone morphogenetic protein 9, epidermal growth factor, hepatocyte growth factor, and platelet-derived growth factor [30].

## 4. Consideration on Age, Species, and Pathophysiological Conditions of Starting Organs

For the generation of an optimal starting matrix for whole organ bioengineering, a careful evaluation should be addressed to the general characteristics of the heart to be decellularized.

While most of the decellularization approaches were applied to adult tissue and organs [7, 25, 27, 33], few attempts have been dedicated to generate acellular young hearts with the rationale for a future development of whole bioengineered equivalents for cardiac transplantation in pediatric patients. The study by Williams et al. offers a very elegant assessment of the properties of fetal, neonatal, and adult decellularized cardiac extracellular matrices. The biochemical and bioinductive comparisons performed evidenced a progressive maturation of the ECM, directly influencing the proliferation of all cardiac cells, especially the myocytes. Through a proteomic approach, it turned out that biochemical content of the 15 most abundant proteins changed, also dramatically, during developmental process. While collagens I and III increased, the opposite was verified for the types IV, V, and VI. Regarding the elastin development, an increased amount could be appreciated for fibrillin I, while fibrillin II tended to disappear at the adult stage. Fibronectin, periostin, emilin I, and perlecan reduced progressively throughout maturation, differently from laminin, which first appeared during the neonatal period. The fetal and neonatal matrices, rich in fibronectin, periostin, collagen IV, and emilin I, seemed to create the ideal microenvironment for the maintenance of the proliferating phenotype in cardiac myocytes [58].

The insights obtained by these observations could promote new biomimetic strategies for the effective engineering of the adult heart ECM.

As previously observed in other studies optimizing the decellularization methodologies for young organs and tissues, fetal and/or neonatal acellular matrices with preserved architecture are achieved by applying less aggressive approaches in respect to the ones used to generate adult ones. Williams and colleagues demonstrated that the SDS concentration used for mature cardiac organs needs to be reduced by 20 times to obtain a similar decellularization yield (compromise in between effective cell removal and matrix preservation) in fetal hearts, due to the faster solubilization of their immature components [58].

Oberwallner et al. evidenced that, independently from the adopted treatment, decellularized adult human heart tissues retain a pigment, that is, lipofuscin, typically observed in aging subjects. As final product of lipid and protein oxidization, unresolved lipofuscin granules could induce cytotoxicity and immune responses in potential clinical therapies [59].

These are not the unique pathological aspects that are expected to be found in adult aging human organs. Especially in the Western countries, hypercholesterolemia and hypertension are frequently diagnosed even in 30 to 40-year-old subjects. The mean age of current heart donors is around 50, age in which effects of these pathologies are commonly observed, as atherosclerosis in the coronary arterial tree or high vascular resistance [3]. A worse pathophysiological scenario is predictable for a heart previously subjected to myocardial infarction (scarring areas rich of fibrotic tissue). As demonstrated recently [59], infarcted donor hearts are not suitable for an effective whole organ engineering strategy because, evidently, their compromised histopathological architecture could not be reversed by the decellularization treatment.

Due to the shortage of human donors and ageing-related pathophysiological signs, animal organs might represent a future, unlimited source for therapeutic strategies based on whole bioengineered organs. A comparison of the results achieved in the decellularization of human (adult, structurally normal) and porcine (relatively young, healthy animals) hearts evidenced the high similarities of the myocardial ECM distribution in the two species [59].

## 5. Cell Seeding Strategies

Several regenerative medicine strategies for the acute and chronic failing hearts have been based on the administration of cells with different phenotypes. In principle, cell infusion or injection was aimed at facing the large cardiomyocyte loss that occurred to the heart after ischemic attack. Among the different cell types, extracardiac stem cells and progenitors, as well as differentiated cell types with contractile activity, demonstrated to be particularly appealing for their ease of harvesting. In fact, endomyocardial biopsy represents a very invasive procedure bringing about a relatively low yield in the number of isolated cardiac stem cells.

Bone marrow stem cells and skeletal myoblasts found large application, unfortunately without effective advantages in terms of recovered global heart function [60–63].

### 5.1. Cell Typologies and Differentiation Modalities

The reconstruction of the whole heart requires not only cells but also the reconstitution of several specialized tissues, as the basket wave architecture of the ventricles, and a patent coronary arterial tree and more complex structures, as functional valves and conduction system [64]. Repopulating cells need therefore to possess or acquire a commitment strictly dependent to the physiological specialization of the subregion of the heart to recreate.

In the first whole heart engineering experience, a bioreactor was used to perfuse the rat decellularized hearts through the coronary arterial tree with an oxygenated cell medium at a constant flow of 6 ml/min. A nonenriched population of neonatal cardiomyocytes, obtained from syngeneic rats and containing also cardiac fibroblasts, smooth muscle, and endothelial cells, was selected for cell repopulation of the perfused organs. After repeated injections in the anterior left ventricle for a total of 5–7.5 × 10^7^ cells, about 50% were found in the effluent in the first 20 minutes. Electrical stimulation on the epicardial surface of the seeded ventricle was also realized after 24 hours from injection. Moreover, in close circuit perfusion mode, reendothelialization was attempted by infusing rat endothelial cells (2 × 10^7^) in the patent aorta. The maximum recellularization yield was achieved near the area of ventricle injections, with about 30% cell retention after 8 days of dynamic culture, high viability, and maintenance of cardiovascular phenotypes in terms of contractility and endothelialization. Functional assessment on cross-sectional rings of repopulated hearts submitted to pulsatile flow revealed that the highest contractile force could be generated at 8 days of seeding by applying less than 4 Hz. This latter allowed reaching the 2% of the force developed in native organs [7].

Rat neonatal cardiomyocytes were used likewise to repopulate porcine decellularized cardiac organs by injecting 8-9 × 10^6^ cells in the anterior left ventricular wall between the diagonal branches of the descending artery. Before the injections through the aorta, perfusion of the acellular hearts was established in a commercial whole-organ bioreactor with oxygenated medium and was stopped only for 60 min to ease the attachment of human umbilical cord blood endothelial cells onto the coronary arteries. Pacing was induced by means of electrodes positioned on the midventricular wall. Injected areas appeared again to be more repopulated, and cells were generally viable. A partial endothelial lining was evident in the coronary arteries by histology and multielectrode array confirmed electrical activity up to 200 mV [27].

Apart from differentiated cells obtained from primary culture extracted by native tissues, multipotent stem cells were applied too. Rat decellularized hearts submitted to 1-year long cryopreservation were seeded with peripheral blood progenitors (2 × 10^7^) obtained from dogs. Conditioning was realized in a modified spinner flask bioreactor at 3 ml/min flow for 9 days. Even if cryopreservation induced a reduction in the size of treated decellularized organs, viability was recorded among adhering cells [65].

Reconstitution of functional parenchyma and vasculature is the fundamental goal in whole heart bioengineering strategies. Differentiated cells may require complex culturing *in vitro*, potentially losing their mature properties (de-differentiation). Multipotent stem cells or progenitors have a relatively limited plasticity and may not be able to commit towards bona fide cardiac myocytes, even after conditioning in a cardiopoietic microenvironment (differentiation media, coculture with neonatal cardiac myocytes or *in vivo* contact) [51, 66–70].

With this aim, (epi)genetic reprogramming might foster the development of the next-generation therapy for a broken heart. A cell population with cardiac progenitor features can now be obtained with a specific strategy. The recent development of induced pluripotent stem cells (iPS) has paved the way to countless translational medicine applications [71, 72]. The effective reprogramming of adult somatic cells, that is, dermal fibroblasts or T-lymphocytes, to pluripotency by forced reactivation of the embryonic developmental programs may allow for the generation of virtually all body cells in unrestrained amount, offering in the future more personalized therapies for diseased patients. Particularly for the cardiovascular field, modeling of genetic cardiac diseases and heart tissue engineering have been made feasible *in vitro* thanks to this technological advancement [73, 74]. Several protocols have been developed either to generate such pluripotent cells (transfection with retroviral vectors, Sendai RNA virus, etc.) or to magnify their differentiation towards the cardiogenic lineage (growth factors cocktails, mechanical conditioning, etc.). Obtained progenitors are currently tested in preclinical approaches of *in vivo* stem cell therapy and tissue engineering [75, 76]. A recent experimental study in an immunosuppressed xenogeneic model coupled this promising tool with another nanotechnological development, that is, thermoresponsive biomaterials. Human iPS-derived cardiac myocytes were seeded onto these specially treated culture dishes, whose hydrophobic plastic surface modifies to hydrophilic state simply by decreasing the temperature from 37 to 20°C. Such a temperature change induces the detachment of the cell layer/s without disrupting the just formed intercellular junctions, particularly important in the electromechanical coupling among cardiomyocytes. Functional cardiac sheets generated with this technique were transplanted into chronically infarcted porcine hearts, inducing an efficient and stable recovery in LV global function after only two months of observation [77, 78].

These highly positive results might clash however with some general and yet unsolved technical issues. Anyway, it must be considered that in respect to the similarly plastic embryonic stem cells (ES), iPS are free from ethical concerns since they are derived from adult tissues. They can be generated from the cells of a patient, with known clinical history and above all preserving his/her integral genetic background [79]. Potentially, an *in vitro* genetic correction might render feasible the reverse of an unhealthy condition to a normal phenotype. An autologous clinical treatment based on so-engineered, patient's cells may presumptively restore the lost function with no immunogenic hazard. Nevertheless, any conserved pluripotent ability after pushed differentiation may expose *in vivo* to uncontrollable teratogenicity if the commitment towards the mature cell of interest has been undertaken incompletely. Other aspects may hamper cell therapies based on iPS-derived cardiomyocytes, as for example, a difficult enrichment of a selected population.

Particularly for the generation of whole bioengineered hearts, pluripotent stem cells are exceptionally attractive. Even though the differentiation of these cells into cardiovascular cells has to follow a long and complicated molecular route [80], ES and iPS have found several applications to reconstruct the vasculature and parenchyma of decellularized hearts. Among the first studies, Ng et al. applied an Activin A and BMP4-based cocktail to differentiate human ES, expressing EGFP under the promoter of the embryonic marker Oct3/4, into multipotent cardiovascular progenitors expressing the lineage marker Nkx 2.5, the homeobox protein goosecoid, the endothelial elements platelet-derived growth factor alpha, vascular endothelial growth factor receptor 2, and E-cadherin. These mesendodermal cells were infused through the aorta of decellularized rat hearts, statically conditioned for 14 days. Undifferentiated ES were also applied to perform a comparison on the ability of transdifferentiation. Decellularized cardiac ECM offered to the seeded cells a microenvironment providing cues for their proper differentiation. In fact, after only 10 days, EGFP positivity was no more detectable in the ECM seeded with undifferentiated ES, thus rendering evident the loss of the expression of stem cell markers, as further demonstrated by gene expression studies. Cardiovascular differentiation was achieved for both cell lines with extensive positivity for Nkx2.5 and cTnT, even if expression of typical cardiomyocyte myosin markers, that is, MyH6, Myl7, and Myl2, was differential between the two populations [81].

In 2013, Lu and coworkers equally demonstrated that it was possible to achieve cell differentiation by the direct contact of the cardiac ECM with pluripotent stem cells, in this case induced ones. Human iPS were previously submitted to *in vitro* differentiation into cardiovascular progenitors by applying Activin A, BMP4, VEGF A, and Dickkopf homologue 1 (DKK1), administered in the cell culture of embryoid bodies with a precise timing. FACS analysis for KDR revealed that a commitment was achieved for nearly 70% of treated iPS. Differentiated cells were seeded into decellularized murine hearts, previously functionalized with either VEGF A/DKK1 to enhance cardiac cell maturation or VEGF A and bFGF to foster revascularization. An amount of 1 × 10^7^ cardiovascular progenitors was infused into decellularized heart scaffolds through the aorta. Conditioning with growth factors was periodically applied after cell seeding to enhance cell differentiation. After 7 days of semidynamic culturing, the highest cell retention was assessed at 10–15%, directly influencing the ability of engrafted cells to exert an electrical activity. Nevertheless, engrafted cells were able to electrically couple, as demonstrated by calcium transients, and sustained electrocardiogram-like signals. Also, the evaluation of the phenotypic fate of engrafted cells confirmed the acquisition of cardiomyocyte differentiation markers, as cTnT, connexin 43, and sarcomeric alpha-actinin. Classical smooth muscle and endothelial proteins, that is, smooth muscle myosin heavy chain, CD31, and VE-cadherin, were found expressed by cells populating the vascular ECM scaffolding of the decellularized hearts.

The acellular natural scaffolds demonstrated hence to possess the ability to inform cardiovascular progenitors for their further differentiation into mature-like cardiac cells, differently from what could happen in the similarly 3D microenvironment of the embryoid bodies. The initial functionalization of the scaffolds with growth factors and their further administration in dynamic culture turned out to boost this process. As a further demonstration, repopulated hearts were also able to show a chronotropic response upon stimulation with isoproterenol, as well as calcium instabilities, reminding of long QT 2 syndrome arrhythmogenicity, after administration of E4031, a selective blocker of the HERG potassium channels [82].

More recently, Ott and his group proved that it was possible to maintain functional and viable constructs of whole decellularized human hearts repopulated with cardiovascular progenitors derived from human iPS for 120 days [33]. The cardiovascular commitment of used pluripotent stem cells was achieved by fine modulation of the Wnt pathway, similarly realized during cardiac embryogenesis for the induction of the mesoderm and the specification of the heart fields. In fact, its upregulation during pluripotent stem cell cardiac differentiation is required during the phases of mesodermal progenitor differentiation in nascent-precardiac and cardiac mesoderm. Contrariwise, active Wnt pathway must be switched off during the fate determination of the cardiac cells in the first or secondary heart fields [80, 83, 84]. The stimulation with Activin A and BMP4, also in combination with VEGF A and DKK1, has a variable ability to induce cardiac commitment in treated iPS, depending especially on the cell lines and the experimental conditions [85, 86]. It is possible to obtain a high yield of differentiation in cardiomyocytes if pluripotent stem cells are submitted to biphasic conditioning with inhibitors of, respectively, glycogen synthase kinase and Wnt. CHIR99021 has been applied to repress the GSK3 pathway, while inducible shRNA of *β*-catenin or alternatively, IWR were used to inhibit Wnt signaling [87, 88]. The fine tuning of these fundamental pathways for cardiac cell fate might bring about 98% of functional cardiomyocytes.

Guyette and colleagues applied CHIR99021 and IWR4 to induce a robust cardiomyocyte differentiation (nearly 85%) of human iPS. Unsorted differentiated cells (500 × 10^6^) were injected into the left ventricle myocardial ECM in the region comprising the LAD and the left circumflex coronary artery. After 3-4 hours of static conditioning, repopulated hearts were exposed to a flow of initially 20 ml/min and then 60 ml/min. Engraftment was evident in injected areas, with a repopulation of 50% of these volumetric regions at 14 days. Cells were positive for myosin heavy chain, sarcomeric alpha-actinin, and cTnT. Electrical activity was demonstrated upon electrode stimulation at 0.8 Hz with a force generation of 350 *μ*N. Notwithstanding the previous cell conditioning with CHIR99021 and IWR4 and the direct contact with the decellularized cardiac ECM, part of the cardiomyocytes displayed signs of immaturity after 14 days. Moreover, while the coronary arterial tree was patent and conveyed oxygenation and nutrients through the perfused medium, no information was disclosed regarding the reconstruction of the vasculature tissue by engrafted cells [33].

Direct cell reprogramming has been proposed as a valid option to apply for the repopulation of the whole heart. Several strategies have been attempted to convert fibroblasts into cardiomyocytes, not with univocal results [89–91]. In 2010, Ieda and colleagues demonstrated that lineage conversion of cardiac fibroblasts in beating myocytes was feasible *in vitro* and *in vivo* by stimulation with a cocktail of retroviral vectors carrying the three genes Gata4, Mef2c, and Tbx5, key transcription factors during the embryonic development of the heart. Without any passage through a pluripotent state, differentiated cells were induced to switch their lineage. *In vitro* overexpression of the three cardiac cell-specific genes was demonstrated to convert the 20% of heart fibroblasts into cardiomyocytes [89]. The relatively low yield of induced transdifferentiation was however not confirmed by others [90].

In 2012, Eulalio et al. identified through a high-throughput functional screening a class of miRs able to stimulate proliferation of neonatal and adult cardiac myocytes [91]. In particular, has-miR-590 and has-miR-199a were demonstrated to possess the ability to induce cardiac regeneration in a murine model of myocardial infarction [91].

Induced cardiomyogenesis, without a passage through a pluripotent stage, has been therefore indicated as the possible way to overcome the limitations related to the use of iPS, namely, relatively low efficiency of reprogramming and differentiation approaches, possible teratogenesis provoked by undifferentiated pluripotent stem cells, and inability to integrate and survive in the injected cardiac ischemic tissue [92–94].

Nevertheless, in the attempt to obtain a viable and working myocardium and generally the other specialized structures of the heart, the reconstruction of the cardiac unit is pivotal [95]. The cardiac unit represents, in fact, the building block of the heart tissue and is comprised of different cell elements, that is, cardiomyocytes, capillaries, and fibroblasts, in a species-specific proportion. A functional cardiac unit is required to maintain tissue homeostasis, while during the onset of pathological conditions, it results to be unbalanced [95].

For effective whole heart reconstruction, it is therefore crucial to rely on a recellularization strategy based on a mixed population of differentiated cells or alternatively, on cardiovascular progenitors with the potential to differentiate in all cardiac cells. As previously evidenced, the decellularized cardiac matrix is a potent inductor of cardiac differentiation of pluripotent stem cells and cardiovascular progenitors [59, 81, 82].

### 5.2. Cell Infusion Approaches

Apart from the cell type and the differentiation strategy applied, another important variable influencing the degree of engraftment and reconstruction of the heart organ is represented by the injection approach.

Direct cell infiltrations of the decellularized anterior left ventricle wall with cardiac myocytes, with or without endothelial cell infusion through the aorta into the coronary arteries, were able to generate variously repopulated areas (max 50%) with contractile abilities and force generation both in small rodent and large animal hearts [7, 25, 27, 33, 65].

Also, the sole retrograde perfusion of the decellularized aorta has been applied to infuse cells for repopulation purposes [81, 82]. Robertson et al. optimized the reendothelialization of rat decellularized whole heart vasculature by infusing endothelial cells, obtained by the rat aorta, in the inferior vena cava and the brachiocephalic artery. This injection modality resulted to be superior to the aortic retrograde cell infusion in terms of cell attachment yield and also of the prevention of thrombogenicity *in vivo*, as well as of the generation of contractility after sequential seeding with neonatal cardiomyocytes [96].

### 5.3. Bioreactors for the Conditioning of Bioengineered Hearts

Post cell seeding organ conditioning is directly influencing the acquisition of appropriate tissue engraftment and maturation. Apart from infrequent cases [81], stimulation is generally provided to the cell-seeded acellular cardiac organs. Ad hoc developed bioreactors or commercial options are adopted for the opportune setting of specific temperature (37°C), provision of oxygen and nutrients, and gas exchange into the whole organ through its coronary arterial tree via the cannulated aorta. Ideally, the seeded heart should be submitted to the same regional blood flow parameters (speed, shear stress, pulsatility, etc.) naturally occurring in the human cardiac organ [97].

However, cell loss, especially during the first hours/days after seeding, represents a concrete concern. In order to prevent washout of seeded elements, several flow settings have been therefore applied, for example, alternated cycles of reperfusion-static conditioning or progressively growing flow [7, 33, 82].

In addition, biochemical, biomechanical, and/or electrical stimuli should be provided.

Preconditioning of the heart ECM with cell medium used for seeding and administration of growth factors during organ perfusion have been demonstrated to ease adhesion and differentiation of infused/injected cells towards more mature cardiac phenotypes [33, 82]. Constant electrical stimulation proved to exert similar effects in terms of cell maturation in tissue-engineered heart constructs [98, 99], as well as in repopulated hearts [7, 27, 33]. Hülsmann et al. proposed in 2013 an automated whole heart bioreactor able to induce a controlled 3D stretching of the left ventricle, by means of an inflatable latex balloon positioned in the ventricular chamber [100]. After 3-4 days of biomechanical stimulation, decellularized rat hearts seeded with C2C12 murine myoblasts showed increased 3D spatial alignment to the fibres of the ECM in respect to the nonstimulated ones, even if cell viability was reduced [100].

A valid bioreactor for the reconstruction of the whole heart, as well as of other organs, requires specific characteristics. As the Langendorff system described before, it has to be composed of several components, among the chief ones a peristaltic pump, an oxygenation system, an air trapping system, flow and/or pressure controllers, biochemical and fluid dynamics biosensors, lodging chambers, and inflow and outflow tubes. In particular, its subparts in contact with the stimulated organ, comprised mounted biosensors, must be realized in materials resistant to corrosion and damage, as well as to terminal sterilization. Alternatively, they must be configured with sterile disposable units, easy to be exchanged for each organ to be conditioned.

Hence, the ideal bioreactor has to be fully compatible with a clinical grade application.

Automation and controllability of the bioreactor operations appear to be crucial for the reproducibility of results. LabView software has been applied in several experiments to control the timing of decellularization, recellularization, and organ conditioning [24, 33, 101].

## 6. Functional Analyses Performed on Whole Bioengineered Hearts

Langendorff apparatus can also be employed after cell seeding to investigate *in vitro* and/or *ex vivo* the degree of maturation and global function of the repopulated heart. In particular, it renders possible the assessment of the tissue-engineered equivalent in terms of contractility, heart rate, cardiac metabolism, and electromechanical coupling.

The study of the newly developed contractile performance, thickness, and telesystolic and telediastolic volumes is usually performed by applying specific techniques, as the insertion of a balloon in the left ventricular chamber or echocardiographic analysis [16, 17].

Regarding *in vivo* functional analyses, transplantation models are the most effective to test the performance of the reconstructed whole hearts. Ng et al. implanted decellularized hearts statically seeded with ES, either differentiated to mesendodermal cells or undifferentiated, in the subcutaneous tissues of SCID mice. In these immunocompromised animal, heart scaffolds seeded with mesendodermal cells revealed a higher vascularization, increased cellularity than the ES-repopulated ones, but similar cardiovascular differentiation propensity, as confirmed by immunodetection of cTnT, CD31, and Nkx2.5 [81].

A heterotopic transplantation model in athymic rats was chosen by Robertson and colleagues. They implanted the regenerated hearts by means of two anastomoses: the donor's heart aorta and left pulmonary artery to the recipient's abdominal aorta and vena cava, respectively. In respect to the nonrepopulated hearts, the rate of clotting in the aorta of seeded scaffolds was significantly lower, as well as the endoventricular cavity was less thrombogenic. Interestingly, both recellularized and acellular scaffolds presented repopulation by endothelial-like cells (CD31 and VEGFR2) [96].

In 2016, Kitahara and colleagues performed the first heterotopic implantation in an allogeneic large animal model. A total of three decellularized porcine heart scaffolds, that is, one unseeded, one with aortic cell infusion of porcine mesenchymal stem cells, and lastly, one with injections in the ventricular wall of the same cell type, were transplanted into three pigs, serving as recipients. Superior vena cava and aorta were considered as the outflow and the inflow of the hearts, while the other vessels were closed by suture. Although the statistical power of the experimental plan is very low (one animal for each of the three conditions tested), the authors reported some observations in line with the data disclosed for the same model in the small rodent. Intraoperative angiography revealed an immediate clotting of the coronary arteries in unseeded hearts, causing a block of blood perfusion, while the same phenomena were prevented by previous *in vitro* cell seeding. In addition, bioengineered hearts displayed thrombosis and inflammatory cell infiltrates too [32].

Heterotopic transplantation might be not enough powerful as a model to test functionality of the reconstructed heart, but due to the state of the art of organ repopulation, it is still premature to move to a more physiological and appropriate orthotopic implantation model.

## 7. Immune Response Issues in Whole Heart Bioengineering

As in cardiac transplantation, the main variable for the success of a whole heart replacement with a bioengineered equivalent is ultimately correlated to the organ acceptance by the immune system of the recipient.

In allogeneic settings, human leukocyte antigens (HLAs) are known in transplantation medicine to induce immune responses towards the donor's implanted tissue (heart, cardiac valves, etc.) [102]. Indeed, nucleic acids might result in similar effects [46, 48, 103]. Decellularization strategies have to ensure not only the preservation of the native extracellular matrix of the donor's tissue/organ but also the full elimination of resident cellular components, comprised HLAs and nucleic acids.

Guyette et al. proved the elimination of HLAs from human heart submitted to decellularization, as verified by immunofluorescence and single antigen bead assay. In addition, they evaluated the immunogenic profile of native and decellularized human myocardium in respect to porcine decellularized one in a rat subcutaneous model. Macrophage infiltration was disclosed as evident for all groups analyzed, with a significantly higher amount of the proregenerative M2 phenotype in the human-rat xenogeneic settings. No significant changes were revealed in the whole blood cell count among the considered groups [33].

The rat subcutaneous model is definitely applied as one primary test for the evaluation of biocompatibility of novel biomaterials. It offers the possibility to verify the immune response generated against the tested material in a relatively easy and short time model. However, for a future clinical application, it is not sufficient to use this sole methodology to test effective biocompatibility in human settings. *In vitro* direct contact assays based on human macrophages should simulate more appropriately the allogeneic interaction to be realized after implantation of a bioengineered heart in a human recipient.

As already mentioned before, other sources of immunogenicity in a human heart scaffold can be retrieved in the presence of ageing-dependent lipofuscin formations [59]. An effective decellularization methodology able to remove these granules has not been conceived yet.

For the whole heart bioengineering strategies based on animal organs, more dramatic immunological responses are to be expected in the case of xenoantigen retention in the decellularized cardiac ECM. The alpha-gal epitope is a sugar residue not metabolized by humans but present in all mammalian tissues so far used to generate bioprostheses [46, 103]. In heart and kidney xenotransplantation models, it induced a hyperacute rejection of the implanted organ [104]. Several strategies have been applied to remove this xenoantigen from animal tissues. The only decellularization methodology that demonstrated so far to completely eliminate the xenoepitope in cardiovascular tissues (porcine heart valves as well as animal pericardia) is TRICOL [46]. Conversely, other methodologies of cell extraction did not show a similar ability [105, 106].

In the case of inefficient xenoantigen removal, a sequential treatment with alpha-galactosidase is the most effective solution to generate alpha-gal-free cardiovascular scaffolds [107]. Moreover, thanks to the application of transgenesis programs, pigs deprived of the alpha-gal expression have been generated [108].

Alpha-gal is surely the most dangerous xenoantigen in xenotransplantation, but it is not the unique one to be involved in immune responses. Sialic acids, for example, Neu5Gc, have been also demonstrated to elicit sustained responses and allergic states [109, 110]. So far, no chemical tissue manipulation has been proven to remove Neu5Gc sugar, while knockout animals for the corresponding gene and alpha-gal have been successfully realized in a transgenic pig line [111].

Interspecies transmission of microorganisms remains an important concern not only for the possible spread of viruses [112, 113] but also for potential contaminations by noninactivated resident bacterial species and spores. Available methodologies for terminal sterilization demonstrate effectiveness for all medical devices, apart from the biological class of decellularized tissues. In fact, their sterilization power is sufficient to remove any microorganism, but they induce important degeneration of the ECM. It is thus mandatory to formulate effective terminal sterilization strategies not affecting the quality of the ECM but abating the bioburden associated to treated tissues (Fidalgo et al. submitted).

Not only the extracellular matrix and donor's residual elements but also cells employed for repopulation could be a target of the immune system reactivity prompted by the recipient. In this perspective, it will be necessary to consider that the only allogeneic cells to be well tolerated in transplantation are the mesenchymal stem cells, which were able to suppress mixed T-lymphocyte reactions, secrete anti-inflammatory factors, and hence often utilized as ancillary elements in hematopoietic cell infusions [114]. However, the lack of a robust cardiomyocyte differentiation potential in these cells renders them not appealing for heart repopulation strategies, as it could be with pluripotent stem cells. Nevertheless, even if iPS can be generated by using somatic cells of the same cardiopathic patient, an evaluation of their immunogenic potential should be carefully addressed to exclude possible adverse alterations introduced during reprogramming and differentiation phases.

## 8. Novel Technologies with Potential Impact on Whole Heart Engineering

Whole heart bioengineering is a continuously evolving multidisciplinary research field, for which optimizations and new technologies are constantly introduced.

Improvements in the phases of decellularization, recellularization, and monitoring of the performance of repopulated hearts allow getting closer and closer to a viable and functional bioengineered equivalent. Several devices for effective and automatized decellularization of the cardiac organ have been realized [29, 101].

For effective monitoring of the repopulation, fluorescence microscopy was utilized. In particular, infused cells were marked with a fluorescent cell tracker in order to follow their fate and distribution after injection [29, 96].

Interestingly, whole heart bioengineering might generate intermediate products with marketable promises. Decellularized myocardial matrices have been transformed in naturally inspired hydrogels rich in collagen, elastin, fibronectin, and glycosaminoglycans [115]. These hydrogels are characterized by self-assembly into a nanoscaffold (40–100 nm fibre diameter). They can be opportunely modified in order to increase the elastic modulus, for example, by incorporation of PEG. They can find application as supports in 2D and 3D cell models *in vitro* to evaluate pharmacological effects on seeded cardiac stem cells, either native or induced [35, 116]. In addition, they demonstrate to offer a suitable microenvironment for cell engraftment and neovascularization in preclinical models of acute myocardial infarction [116].

## 9. Outstanding Questions Yet to Be Replied for Clinical Translation

Many questions remain still to be answered for a potential clinical application of these regenerated hearts.

As first, valid sterilization procedures are still missing not only for decellularized hearts but also for other tissues with inferior tridimensional complexity. Novel treatments have demonstrated efficacy in the decontamination of whole cardiac scaffolds [35]. It will be indeed of paramount importance for clinical application to guarantee terminal sterilization of the decellularized hearts before their repopulation (Fidalgo et al. submitted).

Moreover, technical inabilities are hampering the effective reconstruction of the whole organ. However, while progressing in the evaluation of novel decellularization and recellularization approaches, it will be essential to formulate correct preservation methodologies for these bioengineered organs. Currently applied strategies have been developed for human donor cadaveric tissues (cryopreservation) or for animal-derived analogues (glutaraldehyde fixation), but several drawbacks were evidenced by the routine practice, as reduced or abated cell viability and ECM damages, possibly responsible for their degeneration once *in vivo* [102]. Novel methodologies have been advanced experimentally in the recent years also for decellularized scaffolds [117] (Zouhair et al. in submission).

Hopefully, all these procedures will find soon application in the clinical tissue bank sector and will be optimized for whole engineered organs.

As previously pointed out by Scarritt et al. in a review focused on the challenges of whole organ recellularization, FDA and CE need to provide approval to the whole bioengineered heart for its clinical application, similarly to what happens to other medical devices [118]. Due to its peculiar features, the bioengineered heart might be considered as a combination product, that is, a medical device that comprises more elements, namely, biologic, drug, and/or device, with premarket approval potentially obtained by passing the examination of only one of the FDA and CE responsible offices [118].

In order to assess effectively if the implantation of the bioengineered heart is a safe procedure and could receive the acceptance by the qualified regulatory offices, it will be of paramount importance to select an appropriate cohort of patients to perform an effective safety clinical trial.

## Figures and Tables

**Table 1:** Schematic description of the methodologies applied so far for whole heart decellularization.

Author	Year	Species	Decellularization protocol																
Ott	2008	Rat	10 *μ*M adenosine in heparanized PBS, 15 min, 77.4 mmHg				1% SDS in deionized water, 12 h, 77.4 mmHg				Deionized water, 15 min, 77.4 mmHg			1% Triton X-100 in deionized water, 30 min, 77.4 mmHg			100 IU/ml penicillin-G/streptomycin and 100 IU/ml amphotericin B in PBS, 124 h		

Wainwright	2010	Pig	Freezing, 16 h, −80°C		Thawing in distilled water, RT	Distilled water, 15 min, 1 l/min, RT	2x PBS, 15 min, 1 l/min, RT	0.02% trypsin, 0.05% EDTA, and 0.05% NaN3, 2 h, 1 l/min, 37°C	Distilled water, 5 min, RT	2x PBS, 15 min, 1 l/min, RT	3% Triton X-100, 0.05% EDTA, and 0.05% NaN3 in distilled water, 2 h, 1.3 l/min, RT	Distilled water, 5 min, RT	2x PBS, 15 min, 1 l/min, RT	4% DCA in distilled water, 2 h, 1.3 l/min, RT	Distilled water, 5 min, RT	2x PBS, 15 min, 1 l/min, RT	0.1% peracetic acid and 4% ethanol, 1 h, 1.7 l/min	PBS, 15+ 15 min, 1.7 l/min	Distilled water, 15+ 15+ 15 min, 1.7 l/min

Weymann	2011	Pig	4% SDS in PBS, 3 h, 2 l/min, 100 mmHg, 37°C			PBS, 15 min, 37°C	4% SDS in PBS, 3 h, 2 l/min, 100 mmHg, 37°C		PBS, 15 min, 37°C	4% SDS in PBS, 3 h, 2 l/min, 100 mmHg, 37°C		PBS, 15 min, 37°C	4% SDS in PBS, 3 h, 2 l/min, 100 mmHg, 37°C		PBS, 24 h, 1.5 l/min				
	2014														100 *μ*g/ml penicillin/streptomycin in PBS, 24 h, 1.5 l/min				

Akhyari	2011	Rat	I	10 mM adenosine in heparanized PBS, 15 min, 77.5 mmHg			1% SDS in deionized water, 12 h, 77.5 mmHg			Deionized water, 15 min, 77.5 mmHg			1% Triton X-100 in deionized water, 30 min, 77.5 mmHg			100 IU/ml penicillin-G/streptomycin and amphotericin B, 124 h			
			II	0.025% trypsin and 0.05% EDTA in PBS, 1 h, 77.5 mmHg, 37°C		Deionized water, 15 min		3% Triton X-100 in deionized water, 1 h, 77.5 mmHg			Deionized water, 15 min		4% DCA in deionized water, 1 h, 77.5 mmHg, RT			Deionized water, 15 min, RT		0.1% acetic acid in deionized water, 1 h	
			III	20% glycerol, 0.05% NaN3, and 25 mM EDTA in 0.9% NaCl, 3 d, 77.5 mmHg			4.2% DCA and 0.05% NaN3 in deionized water, 3 d, 77.5 mmHg			20% glycerol, 0.05% NaN3, and 25 mM EDTA in 0.9% NaCl, 2 d, 77.5 mmHg			1% SDS and 0.05% NaN3 in deionized water, 2 d, 77.5 mmHg			3% Triton X-100 and 0.05% NaN3 in deionized water, 2 d, 77.5 mmHg			100 IU/ml penicillin-G/streptomycin, 12 h
			IV	10 mM adenosine in heparanized PBS, 15 min, 77.5 mmHg		1% SDS, 1% DCA, and 0.05% NaN3 in deionized water, 12 h, 77.5 mmHg		Deionized water, 15 min, 77.5 mmHg	20% glycerol, 0.05% NaN3, and 25 mM EDTA in 0.9% NaCl, 12 h, 77.5 mmHg		Deionized water, 15 min, 77.5 mmHg	1% Saponin and 0.05% NaN3 in deionized water, 12 h, 77.5 mmHg		Deionized water, 15 min, 77.5 mmHg	20% glycerol, 0.05% NaN3, and 25 mM EDTA in 0.9% NaCl, 12 h, 77.5 mmHg		Deionized water, 15 min, 77.5 mmHg	200 IU/ml Dnase I and 50 ml/mol MgCl in PBS	

Aubin	2013	Rat	10 mM adenosine in heparanized PBS, 15 min, 77.5 mmHg			1% SDS, 1% DCA, and 0.05% NaN3 in deionized water, 12 h, 77.5 mmHg		Deionized water, 15 min, 77.5 mmHg	20% glycerol, 0.05% NaN3, and 25 mM EDTA in 0.9% NaCl, 12 h, 77.5 mmHg		Deionized water, 15 min, 77.5 mmHg	1% Saponin and 0.05% NaN3 in deionized water, 12 h, 77.5 mmHg		Deionized water, 15 min, 77.5 mmHg	20% glycerol, 0.05% NaN3, and 25 mM EDTA in 0.9% NaCl, 12 h, 77.5 mmHg		Deionized water, 15 min, 77.5 mmHg	200 IU/ml Dnase I and 50 mM MgCl in PBS	100 IU/ml penicillin/streptomycin in PBS, 12 h, 4°C

Remlinger	2012	Pig	Freezing, 24 h, −80°C		Thawing in deionized water, ON, 4°C	Deionized water, 15–25 min, 400 ml/min	2x PBS, 15 min, 700 ml/min	Deionized water, 10 min, 750 ml/min	0.2% trypsin, 0.05% EDTA, and 0.05% NaN3, 3 h, 1200–1800 ml/min, 37°C	Deionized water, 10 min, 1900 ml/min, RT	2x PBS, 10 min, 1950 ml/min, RT	3% Triton X-100, 0.05% EDTA, and 0.05% NaN3, 2.5 h, 2000–2100 ml/min, RT	Deionized water, 10 min, 2150 ml/min, RT	2x PBS, 10 min, 2180 ml/min, RT	4% SDC, 3 h, 2200 ml/min, RT	Deionized water, 15 min, 2200 ml/min, RT	2x PBS, 15 min, 2200 ml/min, RT	Deionized water, 5 min, 750 ml/min, RT	1x PBS, 5 min, 1500 ml/min, RT

Merna	2013	Pig	I	Freezing, 24 h, −80°C	Thawing, RT	0.02% Trypsin, 0.05% EDTA, and 0.05% NaN3, 3 d						3% Triton X-100, 0.05% EDTA, and 0.05% NaN3, 4 d							
			II	Freezing, 24 h, −80°C	Thawing, RT	0.02% Trypsin, 0.05% EDTA, and 0.05% NaN3, 1 d		3% Triton X-100, 0.05% EDTA, and 0.05% NaN3, 6 d											
			III	Freezing, 24 h, −80°C	Thawing, RT	0.02% Trypsin, 0.05% EDTA, and 0.05% NaN3, 7 d													
			IV	Freezing, 24 h, −80°C	Thawing, RT	3% Triton X-100, 0.05% EDTA, and 0.05% NaN3, 7 d													

Methe	2014	Pig	Washed in 1% penicillin/streptomycin and 1% amphotericin B		Freezing in PBS, 24 h, −80°C	Thawing, RT	Washed in distilled water	Short perfusion of 4% SDC^∗^	Immersion and agitation in 4% SDC, 6 h, 37°C^∗^		Perfusion with 1% SDC, 6 h, RT^∗^		Perfusion with distilled water, 6 h, RT^∗^		Perfusion with 1% Triton X-100, 12 h^∗^		Immersion and agitatio in 1% Triton X-100, 12 h^∗^		Perfusion with 0.1% peracetic acid in PBS, 3 h

Momtahan	2015	Pig	Washed in heparin (10 IU/m), 100 IU/ml penicillin, 100 *μ*g/ml streptomycin, and 25 *μ*g/ml amphotericin B		Freezing in heparanized water, −20°C	Thawing, ON, 4°C	1x PBS, 1 h, 0–4 psi, 23°C	Distilled water, 1 h, 5 psi, 23°C		0.5% SDS in distilled water, 2 h, 5 psi, 23°C^∗∗^		Distilled water, 2 h, 5 psi, 23°C^∗∗^		Distilled water, ON, 3 psi, 23°C		1% Triton X-100, 2 h, 3–5 psi, 23°C		Distilled water, 5 h, 5 psi, 23°C	

Guyette	2016	Human	Heparanized PBS (1 IU/ml), 1 h, 60 mmHg, RT				1% SDS in deionized water, 168 h, 60 mmHg, RT				Deionized water, 24 h, 60 mmHg, RT			1% Triton X-100 in deionized water, 24 h, 60 mmHg, RT			PBS, 168 h, 60 mmHg, RT		

Kitahara	2016	Pig	Freezing, 24 h, −80°C			Thawing, 4°C		Deionized water, 15 min, 100 ml/min		1% SDS in deionized water, 3 h, 100 mmHg, 200–1000 ml/min, 37°C^∗∗∗^			Deionized water, 15 min, 1000 ml/min^∗∗∗^		1% Triton X-100 in deionized water, 3 h, 1000 ml/min			Deionized water, 1 h, 1000 ml/min	

Sanchez	2016	Human	1% SDS in deionized water, 60 l in 4 d, 80–100 mmHg							Washed in water, 20 l, 80–100 mmHg					Penicillin/streptomycin in PBS, 10 l, 80–100 mm Hg				

RT: room temperature; EDTA: ethylenediaminetetraacetic acid; NaN_3_: sodium azide; DCA: deoxycholic acid; PBS: phosphate-buffered saline; SDS: sodium dodecyl sulfate; SDC: sodium deoxycholate; psi: pound-force per square inch. ^∗^This group of washes has been repeated 8 times. ^∗∗^This group of washes has been repeated 3 times. ^∗∗∗^This group of washes has been repeated 3 times.

## References

[1] Harvey W. (1957). De Motu Cordis. *The Circulation of the Blood and Other Writings, translated by Kenneth J. Franklin*.

[2] Ambrosy A. P., Gheorghiade M., Chioncel O., Mentz R. J., Butler J. (2014). Global perspectives in hospitalized heart failure: regional and ethnic variation in patient characteristics, management, and outcomes. *Current Heart Failure Reports*.

[3] Cowie M. R. The global burden of heart failure. https://www.escardio.org/static_file/Escardio/Web/Congresses/Slides/Heart%20failure%202015/1183%20-%20The%20global%20burden%20of%20heart%20failure.%20-%20Martin%20COWIE%20(London,%20United%20Kingdom).pdf.

[4] Stehlik J., Bavaria J. E., Bax J. (2016). Heart, lung, and vascular registries: evolving goals, successful approaches, and ongoing innovation. *The Journal of Heart and Lung Transplantation*.

[5] Goldbarg S. H., Elmariah S., Miller M. A., Fuster V. (2007). Insights into degenerative aortic valve disease. *Journal of the American College of Cardiology*.

[6] Singh R. K., Humlicek T., Jeewa A., Fester K. (2016). Pediatric Cardiac Intensive Care Society 2014 consensus statement. *Pediatric Critical Care Medicine*.

[7] Ott H. C., Matthiesen T. S., Goh S.-K. (2008). Perfusion-decellularized matrix: using nature’s platform to engineer a bioartificial heart. *Nature Medicine*.

[8] Taylor D. A. (2009). From stem cells and cadaveric matrix to engineered organs. *Current Opinion in Biotechnology*.

[9] Jung J. P., Bhuiyan D. B., Ogle B. M. (2016). Solid organ fabrication: comparison of decellularization to 3D bioprinting. *Biomaterials Research*.

[10] Badylak S. F., Gilbert T. W. (2008). Immune response to biologic scaffold materials. *Seminars in Immunology*.

[11] Gilbert T. W., Sellaro T. L., Badylak S. F. (2006). Decellularization of tissues and organs. *Biomaterials*.

[12] Crapo P. M., Gilbert T. W., Badylak S. F. (2011). An overview of tissue and whole organ decellularization processes. *Biomaterials*.

[13] Langendorff O. (1895). Untersuchungen am überlebenden Säugetierherzen. *Pfluegers Archiv*.

[14] Yeo J. M., Tse V., Kung J. (2017). Isolated heart models for studying cardiac electrophysiology: a historical perspective and recent advances. *Journal of Basic and Clinical Physiology and Pharmacology*.

[15] Di Lisa F., Menabò R., Barbato R., Siliprandi N. (1994). Contrasting effects of propionate and propionyl-L-carnitine on energy-linked processes in ischemic hearts. *The American Journal of Physiology*.

[16] Döring H. J. (1990). The isolated perfused heart according to Langendorff technique--function--application. *Physiologia Bohemoslovaca*.

[17] Sutherland F. J., Hearse D. J. (2000). The isolated blood and perfusion fluid perfusion heart. *Pharmacological Research*.

[18] Ytrehus K. (2000). The ischemic heart—experimental models. *Pharmacological Research*.

[19] Skrzypiec-Spring M., Grotthus B., Szelag A., Schulz R. (2007). Isolated heart perfusion according to Langendorff—still viable in the new millennium. *Journal of Pharmacological and Toxicological Methods*.

[20] Dijkman M. A., Heslinga J. W., Sipkema P., Westerhof N. (1996). Perfusion-induced changes in cardiac O_2_ consumption and contractility are based on different mechanisms. *The American Journal of Physiology*.

[21] Assayag P., Charlemagne D., Marty I. (1998). Effects of sustained low-flow ischemia on myocardial function and calcium-regulating proteins in adult and senescent rat hearts. *Cardiovascular Research*.

[22] de Bakker J. M., Coronel R., Tasseron S. (1990). Ventricular tachycardia in the infarcted, Langendorff-perfused human heart: role of the arrangement of surviving cardiac fibers. *Journal of the American College of Cardiology*.

[23] Kadipasaoglu K. A., Bennink G. W., Conger J. L. (1993). An ex vivo model for the reperfusion of explanted human hearts. *Texas Heart Institute Journal*.

[24] Akhyari P., Aubin H., Gwanmesia P. (2011). The quest for an optimized protocol for whole-heart decellularization: a comparison of three popular and a novel decellularization technique and their diverse effects on crucial extracellular matrix qualities. *Tissue Engineering Part C: Methods*.

[25] Weymann A., Loganathan S., Takahashi H. (2011). Development and evaluation of a perfusion decellularization porcine heart model—generation of 3-dimensional myocardial neoscaffolds. *Circulation Journal*.

[26] Wainwright J. M., Czajka C. A., Patel U. B. (2010). Preparation of cardiac extracellular matrix from an intact porcine heart. *Tissue Engineering Part C: Methods*.

[27] Weymann A., Patil N. P., Sabashnikov A. (2014). Bioartificial heart: a human-sized porcine model – the way ahead. *PLoS One*.

[28] Remlinger N. T., Wearden P. D., Gilbert T. W. (2012). Procedure for decellularization of porcine heart by retrograde coronary perfusion. *Journal of Visualized Experiments*.

[29] Merna N., Robertson C., La A., George S. C. (2013). Optical imaging predicts mechanical properties during decellularization of cardiac tissue. *Tissue Engineering Part C: Methods*.

[30] Methe K., Bäckdahl H., Johansson B. R., Nayakawde N., Dellgren G., Sumitran-Holgersson S. (2014). An alternative approach to decellularize whole porcine heart. *BioResearch Open Access*.

[31] Momtahan N., Poornejad N., Struk J. A. (2015). Automation of pressure control improves whole porcine heart decellularization. *Tissue Engineering Part C: Methods*.

[32] Kitahara H., Yagi H., Tajima K. (2016). Heterotopic transplantation of a decellularized and recellularized whole porcine heart. *Interactive Cardiovascular and Thoracic Surgery*.

[33] Guyette J. P., Charest J. M., Mills R. W. (2016). Bioengineering human myocardium on native extracellular matrix novelty and significance. *Circulation Research*.

[34] Sánchez P. L., Fernández-Santos M. E., Espinosa M. A. (2014). Data from acellular human heart matrix. *Data Brief*.

[35] Pati F., Jang J., Ha D.-H. (2014). Printing three-dimensional tissue analogues with decellularized extracellular matrix bioink. *Nature Communications*.

[36] Badylak S. F. (2002). The extracellular matrix as a scaffold for tissue reconstruction. *Seminars in Cell & Developmental Biology*.

[37] Bissell M. J., Aggeler J. (1987). Dynamic reciprocity: how do extracellular matrix and hormones direct gene expression?. *Progress in Clinical and Biological Research*.

[38] Badylak S. F. (2007). The extracellular matrix as a biologic scaffold material. *Biomaterials*.

[39] LeGrice I. J., Smaill B. H., Chai L. Z., Edgar S. G., Gavin J. B., Hunter P. J. (1995). Laminar structure of the heart: ventricular myocyte arrangement and connective tissue architecture in the dog. *The American Journal of Physiology*.

[40] Borg T. K., Caulfield J. B. (1981). The collagen matrix of the heart. *Federation Proceedings*.

[41] Jacot J. G., Kita-Matsuo H., Wei K. A. (2010). Cardiac myocyte force development during differentiation and maturation. *Annals of the New York Academy of Sciences*.

[42] Ausoni S., Sartore S. (2009). From fish to amphibians to mammals: in search of novel strategies to optimize cardiac regeneration. *The Journal of Cell Biology*.

[43] Zupanc G. K., Stocum D. L. (2015). Regeneration science needs to broaden its focus to understand why some organisms can regenerate and others not. *Regenerative Medicine*.

[44] Godwin J., Kuraitis D., Rosenthal N. (2014). Extracellular matrix considerations for scar-free repair and regeneration: Insights from regenerative diversity among vertebrates. *The International Journal of Biochemistry & Cell Biology*.

[45] Hynes R. O., Naba A. (2012). Overview of the matrisome--an inventory of extracellular matrix constituents and functions. *Cold Spring Harbor Perspectives in Biology*.

[46] Iop L., Renier V., Naso F. (2009). The influence of heart valve leaflet matrix characteristics on the interaction between human mesenchymal stem cells and decellularized scaffolds. *Biomaterials*.

[47] Wu W., Allen R., Gao J., Wang Y. (2011). Artificial niche combining elastomeric substrate and platelets guides vascular differentiation of bone marrow mononuclear cells. *Tissue Engineering Part A*.

[48] Iop L., Bonetti A., Naso F. (2014). Decellularized allogeneic heart valves demonstrate self-regeneration potential after a long-term preclinical evaluation. *PLoS One*.

[49] Di Liddo R., Aguiari P., Barbon S. (2016). Nanopatterned acellular valve conduits drive the commitment of blood-derived multipotent cells. *International Journal of Nanomedicine*.

[50] Bassat E., Mutlak Y. E., Genzelinakh A. (2017). The extracellular matrix protein Agrin promotes heart regeneration in mice. *Nature*.

[51] Iop L., Chiavegato A., Callegari A. (2008). Different cardiovascular potential of adult- and fetal-type mesenchymal stem cells in a rat model of heart cryoinjury. *Cell Transplantation*.

[52] Leri A., Rota M., Hosoda T., Goichberg P., Anversa P. (2014). Cardiac stem cell niches. *Stem Cell Research*.

[53] Parker K. K., Ingber D. E. (2007). Extracellular matrix, mechanotransduction and structural hierarchies in heart tissue engineering. *Philosophical Transactions of the Royal Society of London. Series B, Biological Sciences*.

[54] Smith L., Cho S., Discher D. E. (2017). Mechanosensing of matrix by stem cells: from matrix heterogeneity, contractility, and the nucleus in pore-migration to cardiogenesis and muscle stem cells in vivo. *Seminars in Cell & Developmental Biology*.

[55] Forsten-Williams K., Chu C. L., Fannon M., Buczek-Thomas J. A., Nugent M. A. (2008). Control of growth factor networks by heparan sulfate proteoglycans. *Annals of Biomedical Engineering*.

[56] Holley R., Meade K., Merry C. R. (2014). Using embryonic stem cells to understand how glycosaminoglycans regulate differentiation. *Biochemical Society Transactions*.

[57] Qiang B., Lim S. Y., Lekas M. (2014). Perlecan heparan sulfate proteoglycan is a critical determinant of angiogenesis in response to mouse hind-limb ischemia. *Canadian Journal of Cardiology*.

[58] Williams C., Quinn K. P., Georgakoudi I., Black L. D. (2014). Young developmental age cardiac extracellular matrix promotes the expansion of neonatal cardiomyocytes in vitro. *Acta Biomaterialia*.

[59] Oberwallner B., Brodarac A., Choi Y. H. (2014). Preparation of cardiac extracellular matrix scaffolds by decellularization of human myocardium. *Journal of Biomedical Materials Research, Part A*.

[60] Hatzistergos K. E., Quevedo H., Oskouei B. N. (2010). Bone marrow mesenchymal stem cells stimulate cardiac stem cell proliferation and differentiation. *Circulation Research*.

[61] Schlosser S., Dennler C., Schweizer R. (2012). Paracrine effects of mesenchymal stem cells enhance vascular regeneration in ischemic murine skin. *Microvascular Research*.

[62] Menasché P. (2003). Cell transplantation in myocardium. *Annals of Thoracic Surgery*.

[63] Rangappa S., Makkar R., Forrester J. (2010). Review article: current status of myocardial regeneration: new cell sources and new strategies. *Journal of Cardiovascular Pharmacology and Therapeutics*.

[64] Chien K. R., Domian I. J., Parker K. K. (2008). Cardiogenesis and the complex biology of regenerative cardiovascular medicine. *Science*.

[65] Crawford B., Koshy S. T., Jhamb G. (2012). Cardiac decellularisation with long-term storage and repopulation with canine peripheral blood progenitor cells. *The Canadian Journal of Chemical Engineering*.

[66] Pozzobon M., Bollini S., Iop L. (2010). Human bone marrow-derived CD133+ cells delivered to a collagen patch on cryoinjured rat heart promote angiogenesis and arteriogenesis. *Cell Transplantation*.

[67] Strauer B.-E., Steinhoff G. (2011). 10 years of intracoronary and intramyocardial bone marrow stem cell therapy of the heart. *Journal of the American College of Cardiology*.

[68] Bartunek J., Behfar A., Dolatabadi D. (2013). Cardiopoietic stem cell therapy in heart failure: the C-CURE (cardiopoietic stem cell therapy in heart failURE) multicenter randomized trial with lineage-specified biologics. *Journal of the American College of Cardiology*.

[69] Malliaras K., Makkar R. R., Smith R. R. (2014). Intracoronary cardiosphere-derived cells after myocardial infarction. *Journal of the American College of Cardiology*.

[70] Houtgraaf J. H., Den Dekker W. K., Van Dalen B. M. (2012). First experience in humans using adipose tissue-derived regenerative cells in the treatment of patients with ST-segment elevation myocardial infarction. *Journal of the American College of Cardiology*.

[71] Takahashi K., Okita K., Nakagawa M., Yamanaka S. (2007). Induction of pluripotent stem cells from fibroblast cultures. *Nature Protocols*.

[72] Nakagawa M., Koyanagi M., Tanabe K. (2007). Generation of induced pluripotent stem cells without Myc from mouse and human fibroblasts. *Nature Biotechnology*.

[73] Kang T. Y., Hong J. M., Kim B. J., Cha H. J., Cho D. W. (2013). Enhanced endothelialization for developing artificial vascular networks with a natural vessel mimicking the luminal surface in scaffolds. *Acta Biomaterialia*.

[74] Brown M. E., Rondon E., Rajesh D. (2010). Derivation of induced pluripotent stem cells from human peripheral blood T lymphocytes. *PLoS One*.

[75] Rufaihah A. J., Huang N. F., Jamé S. (2011). Endothelial cells derived from human iPSCS increase capillary density and improve perfusion in a mouse model of peripheral arterial disease. *Arteriosclerosis, Thrombosis, and Vascular Biology*.

[76] Tulloch N. L., Muskheli V., Razumova M. V. (2011). Growth of engineered human myocardium with mechanical loading and vascular coculture. *Circulation Research*.

[77] Kawamura M., Miyagawa S., Miki K. (2012). Feasibility, safety, and therapeutic efficacy of human induced pluripotent stem cell-derived cardiomyocyte sheets in a porcine ischemic cardiomyopathy model. *Circulation*.

[78] Kawamura M., Miyagawa S., Fukushima S. (2013). Enhanced survival of transplanted human induced pluripotent stem cell-derived cardiomyocytes by the combination of cell sheets with the pedicled omental flap technique in a porcine heart. *Circulation*.

[79] Jung C. B., Moretti A., Mederos y Schnitzler M. (2012). Dantrolene rescues arrhythmogenic RYR2 defect in a patient-specific stem cell model of catecholaminergic polymorphic ventricular tachycardia. *EMBO Molecular Medicine*.

[80] Mummery C. L., Zhang J., Ng E. S., Elliott D. A., Elefanty A. G., Kamp T. J. (2012). Differentiation of human embryonic stem cells and induced pluripotent stem cells to cardiomyocytes: a methods overview. *Circulation Research*.

[81] Ng S. L. J., Narayanan K., Gao S., Wan A. C. A. (2011). Lineage restricted progenitors for the repopulation of decellularized heart. *Biomaterials*.

[82] Lu T.-Y., Lin B., Kim J. (2013). Repopulation of decellularized mouse heart with human induced pluripotent stem cell-derived cardiovascular progenitor cells. *Nature Communications*.

[83] Naito A. T., Shiojima I., Akazawa H. (2006). Developmental stage-specific biphasic roles of Wnt/beta-catenin signaling in cardiomyogenesis and hematopoiesis. *Proceedings of the National Academy of Sciences of the United States of America*.

[84] Ueno S., Weidinger G., Osugi T. (2007). Biphasic role for Wnt/beta-catenin signaling in cardiac specification in zebrafish and embryonic stem cells. *Proceedings of the National Academy of Sciences of the United States of America*.

[85] Kattman S. J., Witty A. D., Gagliardi M. (2011). Stage-specific optimization of activin/nodal and BMP signaling promotes cardiac differentiation of mouse and human pluripotent stem cell lines. *Cell Stem Cell*.

[86] Hazeltine L. B., Simmons C. S., Salick M. R. (2012). Effects of substrate mechanics on contractility of cardiomyocytes generated from human pluripotent stem cells. *International Journal of Cell Biology*.

[87] Wang H., Hao J., Hong C. C. (2011). Cardiac induction of embryonic stem cells by a small molecule inhibitor of Wnt/beta-catenin signaling. *ACS Chemical Biology*.

[88] Lian X., Hsiao C., Wilson G. (2012). Robust cardiomyocyte differentiation from human pluripotent stem cells via temporal modulation of canonical Wnt signaling. *Proceedings of the National Academy of Sciences of the United States of America*.

[89] Ieda M., Fu J.-D., Delgado-Olguin P. (2010). Direct reprogramming of fibroblasts into functional cardiomyocytes by defined factors. *Cell*.

[90] Chen J. X., Krane M., Deutsch M.-A. (2012). Inefficient reprogramming of fibroblasts into cardiomyocytes using Gata4, Mef2c, and Tbx5. *Circulation Research*.

[91] Eulalio A., Mano M., Dal Ferro M. (2012). Functional screening identifies miRNAs inducing cardiac regeneration. *Nature*.

[92] Jayawardena T. M., Egemnazarov B., Finch E. A. (2012). MicroRNA-mediated in vitro and in vivo direct reprogramming of cardiac fibroblasts to cardiomyocytes. *Circulation Research*.

[93] Xu C. (2012). Turning cardiac fibroblasts into cardiomyocytes in vivo. *Trends in Molecular Medicine*.

[94] Kurotsu S., Suzuki T., Ieda M. (2017). Direct reprogramming, epigenetics, and cardiac regeneration. *Journal of Cardiac Failure*.

[95] Ausoni S., Sartore S. (2009). The cardiovascular unit as a dynamic player in disease and regeneration. *Trends in Molecular Medicine*.

[96] Robertson M. J., Dries-Devlin J. L., Kren S. M., Burchfield J. S., Taylor D. A. (2014). Optimizing recellularization of whole decellularized heart extracellular matrix. *PLoS One*.

[97] Muscari C., Giordano E., Bonafè F., Govoni M., Guarnieri C. (2014). Strategies affording prevascularized cell-based constructs for myocardial tissue engineering. *Stem Cells International*.

[98] Schaaf S., Shibamiya A., Mewe M. (2011). Human engineered heart tissue as a versatile tool in basic research and preclinical toxicology. *PLoS One*.

[99] Hirt M. N., Boeddinghaus J., Mitchell A. (2014). Functional improvement and maturation of rat and human engineered heart tissue by chronic electrical stimulation. *Journal of Molecular and Cellular Cardiology*.

[100] Hülsmann J., Aubin H., Kranz A. (2013). A novel customizable modular bioreactor system for whole-heart cultivation under controlled 3D biomechanical stimulation. *Journal of Artificial Organs*.

[101] Kensah G., Gruh I., Viering J. (2011). A novel miniaturized multimodal bioreactor for continuous in situ assessment of bioartificial cardiac tissue during stimulation and maturation. *Tissue Engineering Part C: Methods*.

[102] Iop L., Paolin A., Aguiari P., Trojan D., Cogliati E., Gerosa G. (2017). Decellularized cryopreserved allografts as off-the-shelf allogeneic alternative for heart valve replacement: in vitro assessment before clinical translation. *Journal of Cardiovascular Translational Research*.

[103] Iop L., Gerosa G. (2015). Guided tissue regeneration in heart valve replacement: from preclinical research to first-in-human trials. *BioMed Research International*.

[104] Cooper D. K. C., Ezzelarab M. B., Hara H. (2016). The pathobiology of pig-to-primate xenotransplantation: a historical review. *Xenotransplantation*.

[105] Simon P., Kasimir M. T., Seebacher G. (2003). Early failure of the tissue engineered porcine heart valve SYNERGRAFT in pediatric patients. *European Journal of Cardio-Thoracic Surgery*.

[106] Spark J. I., Yeluri S., Derham C., Wong Y. T., Leitch D. (2008). Incomplete cellular depopulation may explain the high failure rate of bovine ureteric grafts. *The British Journal of Surgery*.

[107] Park S., Kim W.-H., Choi S.-Y., Kim Y.-J. (2009). Removal of alpha-gal epitopes from porcine aortic valve and pericardium using recombinant human alpha galactosidase A. *Journal of Korean Medical Science*.

[108] Choi K., Shim J., Ko N. (2017). Production of heterozygous alpha 1,3-galactosyltransferase (GGTA1) knock-out transgenic miniature pigs expressing human CD39. *Transgenic Research*.

[109] Reuven E. M., Leviatan Ben-Arye S., Marshanski T. (2016). Characterization of immunogenic Neu5Gc in bioprosthetic heart valves. *Xenotransplantation*.

[110] Byrne G. W., Azimzadeh A. M., Ezzelarab M. (2013). Histopathologic insights into the mechanism of anti-non-Gal antibody-mediated pig cardiac xenograft rejection. *Xenotransplantation*.

[111] Salama A., Mosser M., Lévêque X. (2017). Neu5Gc and α1-3 GAL xenoantigen knockout does not affect glycemia homeostasis and insulin secretion in pigs. *Diabetes*.

[112] Kallenbach K., Leyh R. G., Lefik E. (2004). Guided tissue regeneration: porcine matrix does not transmit PERV. *Biomaterials*.

[113] Iop L., Gerosa G. (2013). Cutting-edge regenerative medicine technologies for the treatment of heart valve calcification. *Calcific Aortic Valve Disease*.

[114] van den Akker F., de Jager S. C. A., Sluijter J. P. G. (2013). Mesenchymal stem cell therapy for cardiac inflammation: immunomodulatory properties and the influence of Toll-like receptors. *Mediators of Inflammation*.

[115] Singelyn J. M., DeQuach J. A., Seif-Naraghi S. B., Littlefield R. B., Schup-Magoffin P. J., Christman K. L. (2009). Naturally derived myocardial matrix as an injectable scaffold for cardiac tissue engineering. *Biomaterials*.

[116] Wang R. M., Christman K. L. (2016). Decellularized myocardial matrix hydrogels: In basic research and preclinical studies. *Advanced Drug Delivery Reviews*.

[117] Brockbank K. G. M., Schenke-Layland K., Greene E. D. (2012). Ice-free cryopreservation of heart valve allografts: better extracellular matrix preservation in vivo and preclinical results. *Cell and Tissue Banking*.

[118] Scarritt M. E., Pashos N. C., Bunnell B. A. (2015). A review of cellularization strategies for tissue engineering of whole organs. *Frontiers in Bioengineering and Biotechnology*.

